# Comparative Analysis of the Chloroplast Genome of *Cardamine hupingshanensis* and Phylogenetic Study of *Cardamine*

**DOI:** 10.3390/genes13112116

**Published:** 2022-11-15

**Authors:** Sunan Huang, Zujie Kang, Zhenfa Chen, Yunfei Deng

**Affiliations:** 1 Key Laboratory of Plant Resources Conservation & Sustainable Utilization, South China Botanical Garden, Chinese Academy of Sciences, Guangzhou 510650, China; 2 Management Bureau of Hunan Hupingshan National Nature Reserve, Shimen 415300, China; 3 Center of Conservation Biology, Core Botanical Gardens, Chinese Academy of Sciences, Guangzhou 510650, China

**Keywords:** *Cardamine hupingshanensis*, *Cardamine*, chloroplast genome, molecular markers

## Abstract

*Cardamine hupingshanensis* (K. M. Liu, L. B. Chen, H. F. Bai and L. H. Liu) is a perennial herbal species endemic to China with narrow distribution. It is known as an important plant for investigating the metabolism of selenium in plants because of its ability to accumulate selenium. However, the phylogenetic position of this particular species in *Cardamine* remains unclear. In this study, we reported the chloroplast genome (cp genome) for the species *C. hupingshanensis* and analyzed its position within *Cardamine*. The cp genome of *C. hupingshanensis* is 155,226 bp in length and exhibits a typical quadripartite structure: one large single copy region (LSC, 84,287 bp), one small single copy region (17,943 bp) and a pair of inverted repeat regions (IRs, 26,498 bp). Guanine-Cytosine (GC) content makes up 36.3% of the total content. The cp genome contains 111 unique genes, including 78 protein-coding genes, 29 tRNA genes and 4 rRNA genes. A total of 115 simple sequences repeats (SSRs) and 49 long repeats were identified in the genome. Comparative analyses among 17 *Cardamine* species identified the five most variable regions (*trnH*-GUG-*psbA*, *ndhK*-*ndhC*, *trnW*-CCA-*trnP*-UGG, *rps11*-*rpl36* and *rpl32*-*trnL*-UAG), which could be used as molecular markers for the classification and phylogenetic analyses of various *Cardamine* species. Phylogenetic analyses based on 79 protein coding genes revealed that the species *C. hupingshanensis* is more closely related to the species *C. circaeoides*. This relationship is supported by their shared morphological characteristics.

## 1. Introduction

*Cardamine* Linnaeus is the third largest genus in the family Brassicaceae. The genus comprises approximately 200 species distributed in all continents except Antarctica [1,2,3,4,5,6,7,8]. *Cardamine* was placed in the tribe Arabideae in the past [3], and it is now placed in the tribe Cardmineae on the basis of molecular evidence [4,5,9]. This genus is characterized by basal leaves that are petiolate, rosulate or not, simple and entire, and toothed; racemes that ebracteate or rarely bracteate throughout or only basally, corymbose or in panicles, and elongated in fruit; fruiting pedicels that are slender or thickened, erect, divaricate, or reflexed; petals that are white, pink, purple, or violet; claws that are strongly differentiated from blades or absent, or longer or shorter than sepals. Comprehensive phylogenetic studies of the genus have been considered to be difficult. The main reasons are the diversity of species and multiple events of polyploidization and hybridization within the genus [10,11,12]. Additionally, species belonging to this genus display significant high homogeneity in morphological characters, which makes it difficult to subdivide the genus.

*Cardamine hupingshanensis* (K. M. Liu, L. B. Chen, H. F. Bai and L. H. Liu) is a perennial herb restricted to the neighboring areas between the Hunan and Hupei provinces in China. It is easily distinguished from other species by its simple cauline leaves resembling basal leaves with broadly obovate petals [13,14]. It grows only in cloudy slopes or valleys with coal gangue and running water. Its tender stems and leaves are edible, delicious and nutritious [14,15]. However, the species was assessed as endangered when first published [13], due to its limited populations and excessive harvest by locals. In recent years, the plant has become a research hotspot for its superior selenium enrichment ability. Zhou et al. analyzed the mechanisms of selenium tolerance in *C. hupingshanensis* with transcriptome data from leaves and roots, respectively [16]. Selenium is an essential and beneficial mineral element important to human health. Although high concentrations of selenium are toxic to plants, we still need the organic selenium enriched by plants to supplement this element [17,18]. 

Comparing the genomes of *C. hupingshanensis* and its related species should contribute to further understanding of the genes related to selenium tolerance. It should also enhance our knowledge of the mechanisms of selenium tolerance in plants. However, the systematic position of this particular species remains unknown. Sequencing for this particular species and molecular analysis for the genus *Cardamine* is not only vital for future identification and conservation of the species *C. hupingshanensis*, but also beneficial for finding its related species and revealing the phylogenetic relationships of *Cardamine*. 

Chloroplasts are semi-autonomous organelles in plants, algae and cyanobacteria [19]. The chloroplasts of the higher plants sustain life on Earth through photosynthesis [20]. Despite the fact that the majority of proteins are encoded by a nuclear genome [21], the chloroplast genome (cp genome) plays an essential role in encoding some of the chloroplast proteins, such as the hypothetical chloroplast YCF2 protein [22]. Generally, in the structure of most cp genomes, two identical fragments called inverted repeats (IRs) can be identified, which are separated by one large single copy region (LSC) and one small single copy region (SSC) [23,24,25,26,27,28]. These four regions make up a single quadripartite circular structure about 110 to 170 kb in length [19,29,30,31]. In many evolutionary studies, the cp genomes have been proven to have advantages in lacking recombination events, maternal inheritance, abundance in plants and relatively simple structures [23,26,32,33,34,35,36,37]. The cpDNA regions have been shown to effectively address the genetic variation patterns of some species in *Cardamine* [10,11]. Compared with phylogenetic studies with a few DNA fragments, the cp genome could potentially provide more parsimony-informative sites. So far, more than 8000 complete cp genomes have been published in the NCBI database (https://www.ncbi.nlm.nih.gov/genome/browse#!/organelles/ (accessed on 30 September 2022)). Among them, a total of 22 species of *Cardamine*, to date, have been sequenced and uploaded (Supplementary Table S1). 

In the present study, we sequenced and annotated the complete cp genome of *C. hupingshanensis*. Seventeen available *Cardamine* cp genomes were characterized and compared to further understand the cp genomes of *Cardamine* and identify potential molecular markers to classify *C. hupingshanensis* and its closely related species. A total of 51 species of Brassicaceae were used for the phylogeny investigation based on cp genomes. It is anticipated that the study should determine the systematic position of the plant *C. hupingshanensis* and guide the further study of its selenium tolerance mechanism.

## 2. Materials and Methods

### 2.1. Plant Materials and DNA Extraction

Fresh leaves of *C. hupingshanensis* from multiple individuals were collected from Hupingshan National Natural Reserve (Hunan, China) and dried in silica gel immediately after collection by Prof. Deng Yunfei and Prof. Kang Ming. The voucher specimen (specimen number D27116) was deposited at the herbarium of South China Botanical Garden, Chinese Academy of Sciences (IBSC). The genomic DNA was extracted using a modified cetyl trimethyl ammonium bromide (CTAB) method [38].

### 2.2. Genome Sequencing, Assembly and Annotation

The DNA was sheared to yield approximately 500 bp fragments for library construction. The library preparation was performed using the Nextera XT DNA Library Prep Kit (Illumina) following the manufacturer’s instructions. Illumina Hiseq X Ten instruments at BGI-Wuhan were used to perform paired-end sequencing for each sample. After sequencing, a total of 12,176,144 reads of 150 bp base read length were generated. For the assembly and annotation, the cp genome sequence of *Cardamine parviflora* (accession number: NC_036964) was used as reference. The sequenced clean pair-end reads were filtered and assembled using GetOrganelle v. 1.7.1a [39]. The genome was automatically annotated using Plastid Genome Annotator v. 2019 [40], coupled with manual correction in Geneious v. 11.0.4 [41]. All tRNA genes were further determined using the online tRNAscan-SE service v. 2.0 [42]. The circular cp genome map was created using OGDRAW v. 1.3.1 (http://ogdraw.mpimp-golm.mpg.de/ (accessed on 15 July 2020)) [43]. 

### 2.3. Structural Analysis and Genome Comparison

The annotated protein coding genes of *C. hupingshanensis* were used for the analysis codon usage. The Relative Synonymous Codon Usage (RSCU) was calculated for all codons using CodonW v. 1.4.2 [44]. 

The complete cp genomes of 17 *Cardamine* species were compared using mVISTA v. 2.0 [45] with ShuffleLAGAN mode and the annotation of *C. hupingshanensis* as a reference. DnaSP v. 5.1 [46] was used to calculate the nucleotide variance (Pi) of coding regions and non-coding regions within the 17 *Cardamine* species. IRscope (https://irscope.shinyapps.io/irapp/ (accessed on 3 September 2020)) [47] was used to compare the single copy regions and inverted repeat boundaries in the 17 cp genomes of *Cardamine*. To identify polymorphic SSRs among all *Cardamine* species, the simple sequence repeats (SSRs) were identified for each species using MISA v. 1.0 [48]. The locations and lengths of long repeats (including forward, palindrome, complement, and reverse repeats) were analyzed using REPuter v. 2.74 [49] with the minimum repeat size set to 20 bp. Tandem repeats were determined using Tandem Repeats Finder v. 4.09 [50].

### 2.4. Dataset Construction and Phylogenetic Analysis

A total of 51 complete cp DNA sequences belonging to the family Brassicaceae were obtained from NCBI GenBank database (Table S1). *Tarenaya hassleriana* (Cleomaceae, KX886354) and *Capparis versicolor* (Capparaceae, MH142726) were used as outgroups. For the phylogenetic analysis, the distribution of the 38 species among different tribes in Brassicaceae were as follows: Cardamineae (31), Camelineae (2), Aethionemeae (2), Lepidieae (2), Alysseae (2), Arabideae (2), Brassiceae (3), Cochlearieae (2), Anastaticeae (1), Euclidieae (1), Anchonieae (1) and Sisymbrieae (1). Seventy-nine protein-coding sequences were extracted from the cp genomes and were aligned separately using MAFFT v. 1.3.7 [51]. The alignments were manually examined and adjusted as needed. All alignment genes were concatenated together, resulting in a total length of 71365 bp dataset. 

The substitution models with the best fit were chosen by MrModeltest v. 2.3 (Nylander 2004). RAxML v. 8.0.0 [52] was used to reconstruct the phylogenetic relationship with the maximum likelihood (ML) method. Maximum parsimony (MP) analysis was run in Paup v. 4.0a [53]. Bootstrap values exceeding 50% were shown next to the corresponding branches. Bayesian inference (BI) analysis was conducted using MrBayes 3.2.7 [54] with posterior probabilities (PP) obtained for each branch.

## 3. Results

### 3.1. General Features of the C. hupingshanensis cp Genome

**Figure 1:**
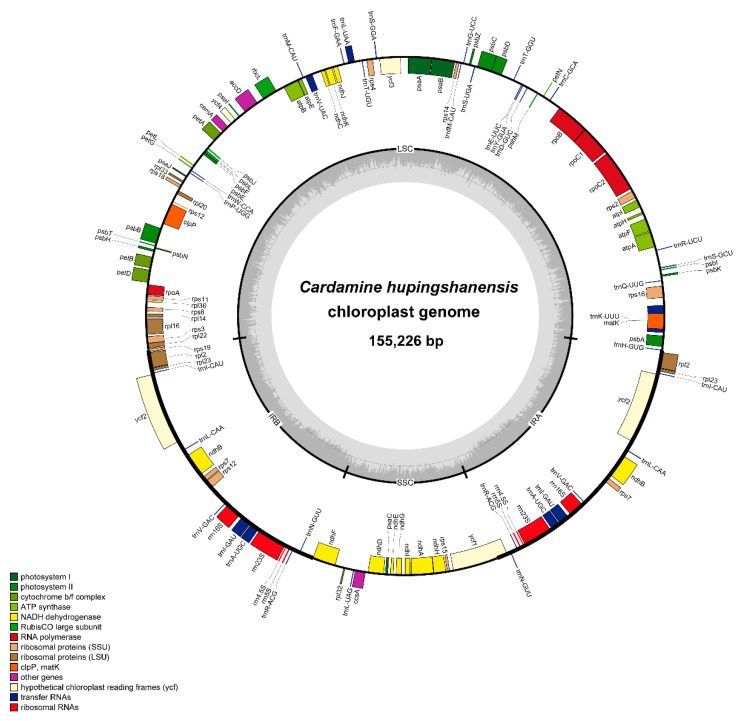
Gene maps of chloroplast genomes of *Cardamine hupingshanensis*. The colored bars indicate known protein-coding genes, tRNA and rRNA. The dark gray area in the inner circle indicates GC content, while the light gray area indicates AT content. LSC, large single copy; SSC, small single copy; IR, inverted repeats.

The complete cp genome of *C. hupingshanensis* is 155226 bp in length with one large single copy region (LSC, 84,287 bp), one small single copy region (17,943 bp) and a pair of inverted regions (IRs, 26,498 bp). A total of 111 unique genes were identified within the *C. hupingshanensis* genome, including 78 protein-coding genes, 29 tRNA genes and 4 rRNA genes. Five protein-coding, seven t-RNA and all four rRNA genes are duplicated because they are located in the IR region. A total of 61 protein-coding genes and 21 tRNA genes are situated in the LSC region, while the SSC region contains 12 protein-coding genes and 1 tRNA gene. Eight protein-coding genes have one intron and three protein-coding genes have two introns (Figure 1, Table 1 and Table 2). Guanine-Cytosine (GC) content makes up 36.3% of the total content. The GC content in IR regions (42.4%) is higher than that of LSC and SSC regions (34% and 29.2%).

### 3.2. Analysis of Codon Bias

Overall, 77,850 bp protein-coding genes were identified in the cp genome of *C. hupingshanensis*, accounting for 50.15% of the entire genome sequence. These genes were encoded in 25,950 codons (Table 3). Of all the codons, Cysteine (Cys, encoded by UGU and UGC, 1.19%) and leucine (Leu, encoded by UUA, UUG, CUU, CUC, CUA and CUG, 10.63%) were the lowest- and highest-frequency amino acids, respectively. The GC content in all protein-coding regions was 37%. The GC content for the first, second and third positions of codons were 39.0%, 34.8% and 37.5%, respectively. Notably, the majority of the preferred codons (RSCU > 1) ended with A or U, with the exception of UUG (RSCU = 1.13).

### 3.3. Comparative Analysis of Genome Structure in Cardamine

Understanding the cp genome differences among species is necessary to measure the species variations. It is also vital to further understand the evolution of the chloroplast. In this study, the complete chloroplast genome of *C. hupingshanensis* was presented, and 16 previously released completed sequences of *Cardamine* species were summarized and shown in Table 2. The smallest genome was in *C. glanduligera* (153,828 bp), and *C. impatiens* (155,611 bp) had the largest cp genome (Table 1). 

**Figure 2:**
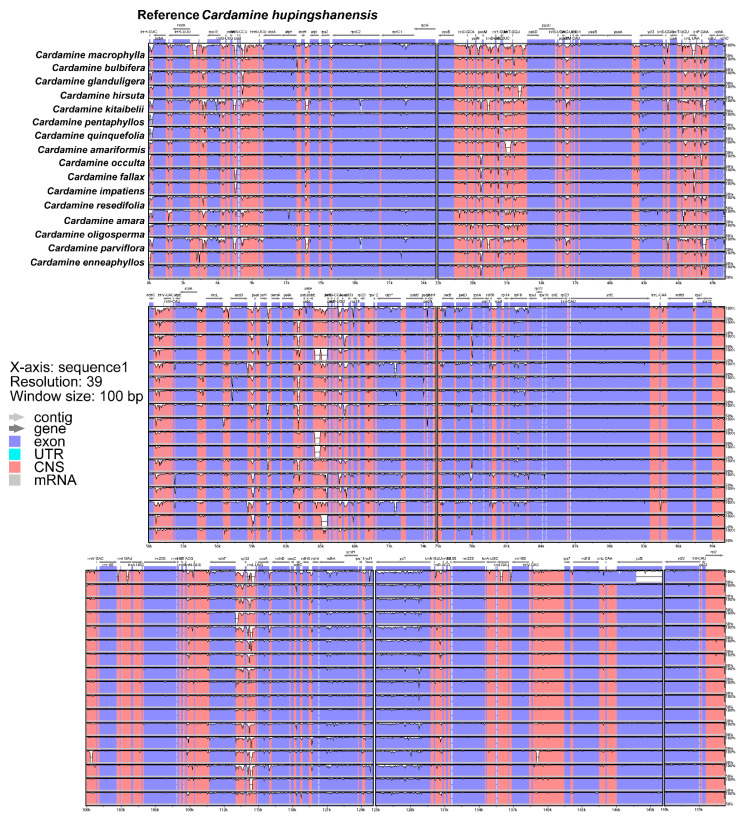
Sequence identity plots of 187 *Cardamine* species using mVISTA. Sequence variations among 17 *Cardamine* species using *C. hupingshanensis* as base reference. Regions in pink indicate conserved non-coding sequences, purple are conserved exons, and white-colored regions identify more variable sites. The Y-axis represents percent identity ranging from 50% to 100%. IR junctions are indicated in parentheses to show LSC, SSC and IR regions.

**Figure 3:**
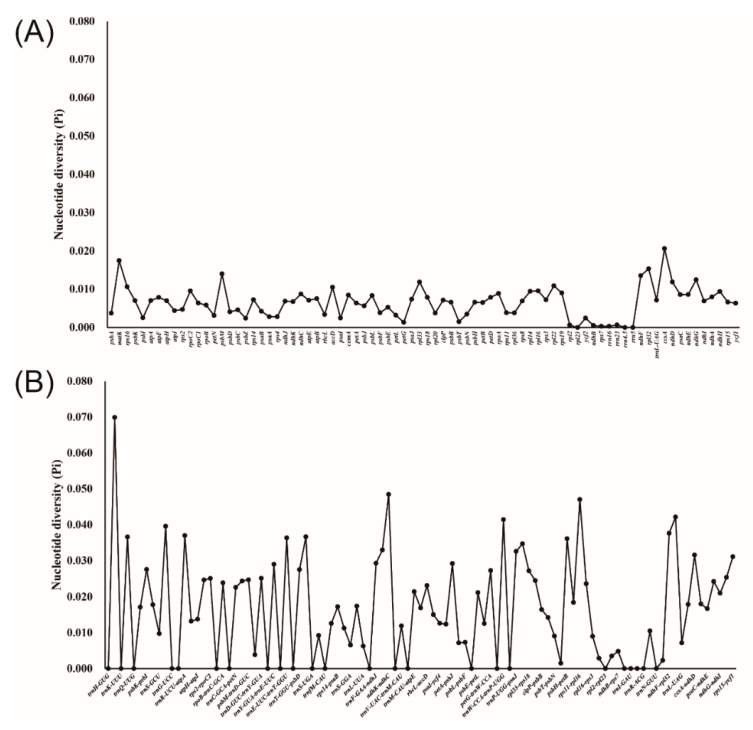
Comparative analysis of the nucleotide diversity (Pi) value among 17 *Cardamine* chloroplast genes. (**A**) Coding regions. (**B**) Non-coding regions.

Comparison of overall cp genome sequence variations in mVISTA showed that the 17 cp genomes within the genus *Cardamine* were highly conserved (Figure 2). Divergence in LSC and SSC regions were higher, whereas variations in IR regions were less discernible. Additionally, among all cp genomes, most divergent regions were apparent in the non-coding regions, particularly the intergenic spacers. The obvious divergent regions in *Cardamine* were the *trnK*-UUU-*rps16*, *psbK*-*trnS*-GCU, *trnY*-GUA-*trnT*-GGU, *ndhC*-*trnV*-UAC, *accD*-*psaI*, *psbE*-*petL*, *ndhF*-*rpl32* and *rpl32*-*trnL*-UAG intergenic spacers. In order to further confirm the sequence variations, the nucleotide variability (Pi) was calculated for the coding and non-coding regions, respectively (Figure 3). Although most of the sequence alignments were rather conserved (Pi < 0.01), five hotspot regions were still found with Pi > 0.04 (*trnH*-GUG-*psbA*, *ndhK*-*ndhC*, *trnW*-CCA-*trnP*-UGG, *rps11*-*rpl36* and *rpl32*-*trnL*-UAG). In the coding regions, 11 protein coding genes (*matK*, *rps16*, *psbM*, *accD*, *rpl33*, *rpl22*, *ndhF*, *rpl32*, *ccsA*, *ndhD* and *ndhG*) were found to be more divergent with Pi > 0.01. 

**Figure 4:**
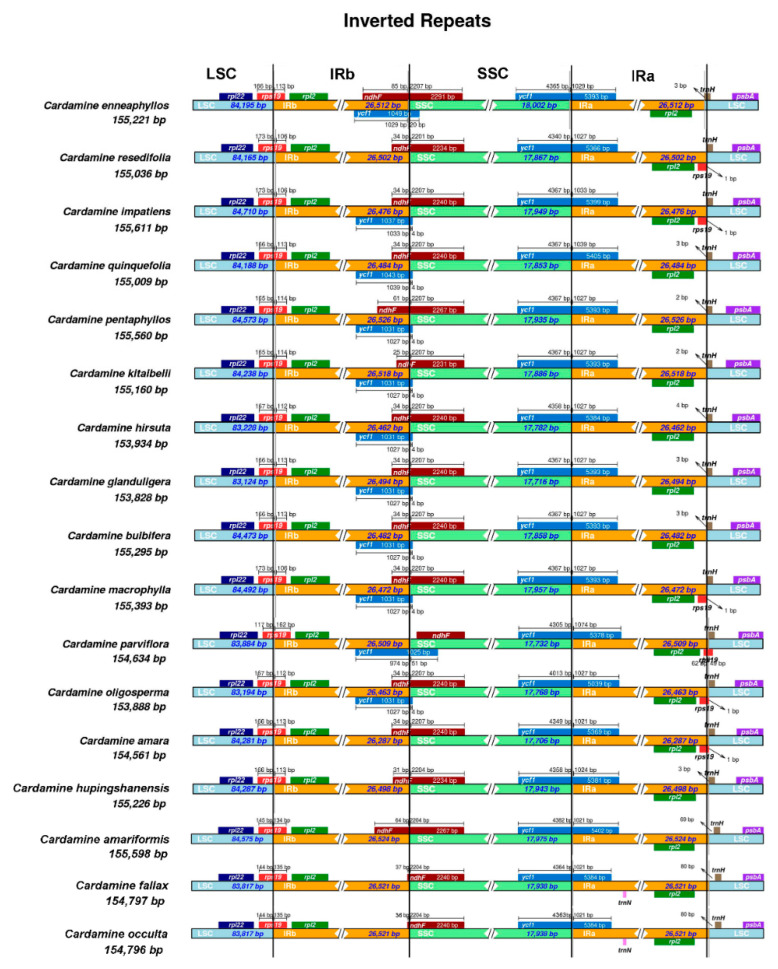
Comparison of the LSC/IRa/SSC/IRb junction among the chloroplast genomes of 17 *Cardamine* species. Colored boxes represent the adjacent border genes. Number above the gene indicates the distance in bp between the ends of genes and the junction sites. The features are not in scale.

Despite the fact that the cp genomes were found to be conserved among *Cardamine* species, some IR contraction and expansion events were still found (Figure 4). Compared to other *Cardamine* species, the IR/SC boundaries in *C. hupingshanensis* were quite conserved. Four genes, *trnH*-GUG, *rps19*, *ycf1* and *ndhF*, are located at the IR-SSC and IR-LSC borders. The positioning of the whole *ndhF* gene in the SSC region was observed only in the genome of *C. parviflora*, while the gene was found to have 25–85 bp in the IRb regions of other species. The *rps19* gene was found to have 162 bp in the IRb region of *C. parviflora*, while the distances were 106–114 bp in other *Cardamine* species. At the junction of SSC/IRa regions, the IRa regions extended 1021–1039 bp into the *ycf1* genes for all analyzed *Cardamine* species except for *C. parviflora* (1074 bp).

### 3.4. Repeat Analysis

Five categories of long repeats (tandem, complement, forward, palindromic and reverse repeats) were detected and analyzed in the cp genome of *C. hupingshanensis*. A total of 75 long repeats, of which 26 were tandem repeats, 1 complement repeat, 17 forward repeats, 28 palindromic repeats and 3 reverse repeats, were identified in the genome. All the repeats ranged in size from 12 to 44 bp. Most of the repeats were between 20 and 29 bp (78.67%), followed by 10–19 bp (14.67%), while 40–49 bp were the least frequent (2.67%). 

**Figure 5:**
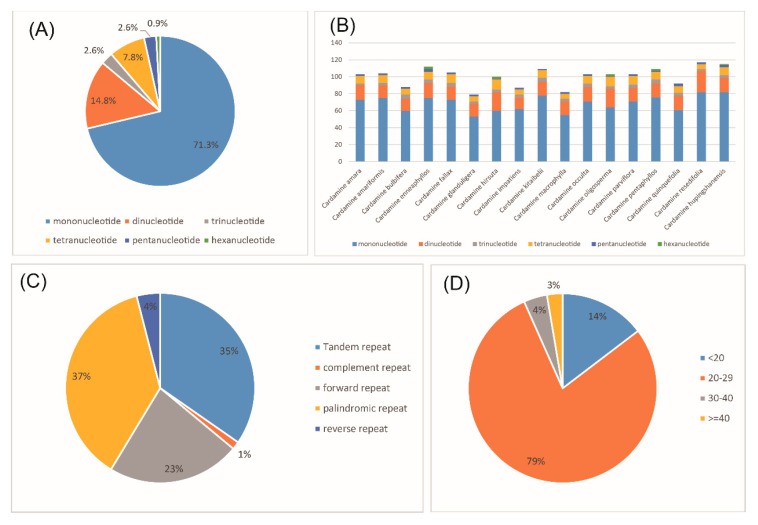
Long repeat and simple sequence repeat (SSR) types and distributions of *C. hupingshanensis* and other species in *Cardamine*. (**A**) Percentage of different SSR types in *C. hupingshanensis*; (**B**) number of SSR types in *Cardamine*; (**C**) number of different long repeat lengths in *C. hupingshanensis*; (**D**) percentage of five long repeat types in *C. hupingshanensis*.

Simple sequence repeats (SSRs) are short stretches of DNA consist of only one, or a few, tandemly repeated nucleotides that distributed throughout the genome. The presence of SSRs is found to be an indicator of mutation hotspots in the genome [23,34,55]. In this study, a total of 115 simple sequences repeats (SSRs) were identified in the cp genome of *C. hupingshanensis*. The number of mono-, di-, tri-, tetra-, penta- and hexanucleotides were 82, 17, 3, 9, 3 and 1, respectively (Supplementary Table S2). Among them, the mononucleotides were the most abundant (71.30%), and the hexanucleotides was the least (0.9%). The exact locations of SSRs in the cp genome are shown in Supplementary Table S3. The corresponding locations were further classified as intergenic spacers (IGS), introns and coding sequences (CDS). The majority of the SSRs were found in the intergenic spacer regions (71.13%) while the coding sequence introns contained the least (12.37%). The SSRs among the cp genomes of the 17 *Cardamine* species were identified and compared (Figure 5). The comparison indicated a high frequency of mononucleotides across all cp genomes (60.00–71.56%). A total of 26 SSRs were identified as polymorphic SSRs among the 17 *Cardamine* species, which could be used as candidate genetic markers for further population genetic studies in the genus *Cardamine* (Table 4).

### 3.5. Phylogenetic Analysis

**Figure 6:**
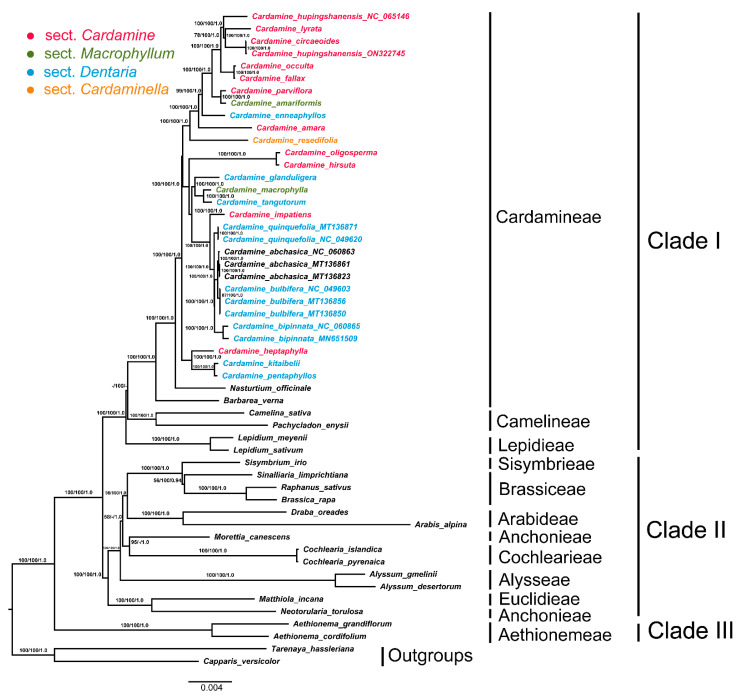
The maximum likelihood (ML) tree of Brassicaceae. Numbers associated with branches are ML bootstrap values, MP bootstrap values and Bayesian posterior probabilities, respectively. Hyphens indicate the bootstrap support or posterior probability lower than 50% or 0.5. *Tarenaya hassleriana* (KX886354) and *Capparis versicolor* (MH142726) were used as outgroups.

The best-fit models for ML and BI analysis were SYM+G. The phylogeny obtained from the MP, ML and BI analyses showed high congruence in tree topologies (Figure 6), which strongly support for the four monophyletic major clades of the family Brassicaceae [BP(MP) = 100%, BP(ML) = 100%, PP = 1.0]. As in previous studies, the tribe Aethionemeae was the basal-most clade in Brassicaceae, followed by diversification of three major evolutionary lineages [56,57,58,59,60]. The tribe Cardamineae was resolved as sister to the clade formed by Camelineae and Lepidieae in clade I. Clade II comprises two tribes: Euclidieae and Anchonieae. Six tribes, Alysseae, Sisymbrieae, Brassiceae, Arabideae, Anastaticeae and Cochlearieae, belonged to clade III, which was identified as sister to clade II with high support [BP(MP) = 100%, BP(ML) = 100%, PP = 1.0]. 

The monophyly of the genus *Cardamine* was strongly supported [BP(MP) = 100%, BP(ML) = 100%, PP = 1.0], in which the genus was divided into three major clades. Clade 1 comprises *C. heptaphylla*, *C. pentaphyllos* and *C. kitaibelii*, being the earliest diverging lineages. Clade 2 containes ten species: *C. oligosperma*, *C. hirsuta*, *C. macrophylla*, *C. glanduligera*, *C. tangutorum*, *C. impatiens*, *C. quinquefolia*, *C. abchasica*, *C. bipinnata* and *C. bulbifera*. Clade 3 includes *C. resedifolia*, *C. amara*, *C. enneaphyllos*, *C. parviflora*, *C. amariformis*, *C. occulta*, *C. fallax*, *C. circaeoides*, *C. lyrata* and *C. hupingshanensis*. *C. resedifolia* was found to be the earliest diverging species in this clade, followed by *C. amara* and *C. enneaphyllos*. The sample *C. hupingshanensis* in the present study was closest to the clade formed by *C. circaeoides* and *C. lyrata*.

## 4. Discussion

### 4.1. Sequence Variations in Cardamine

Sixteen previously released completed sequences of *Cardamine* and the newly sequenced cp genome of *C. hupingshanensis* were used for comparison in this study. No considerable structural rearrangements were detected in the cp genomes of *Cardamine* species. The gene organization, content and order in *Cardamine* genomes appeared to be highly conserved in all seventeen species. Of all analyzed *Cardamine* cp genomes, *C. glanduligera* had the smallest cp genome (153,828 bp) with the smallest LSC region (83,124 bp), and *C. impatiens* had the largest cp genome (155,611 bp) with the largest LSC region (84,696 bp). We assume that the genome size of *Cardamine* species is positively related to the size of the LSC region, and this phenomenon has also been identified in other plant groups [23,26,35,36,37,55]. Codons were shown to have a strong tendency toward A or U at the third codon position, which is similar to the expression of an A/U ending in other plants [23,25,34,55,61]. This phenomenon may explain why the Adenine-Thymine (AT) content is slightly higher than the GC content in all cp genomes of *Cardamine*. 

mVISTA revealed a low divergence between the genomes of the *Cardamine* species, and the IR regions were more conserved than the SC regions. This phenomenon has been found in other angiosperms as well [23,25,26,33,34,36,37,61]. Expansion and contraction events at the junctions of SSR/IR and LSC/IR regions have been recognized as evolutionary signals [23,34,62,63]. In our results, the IR regions are rather conserved in structure. Only one species, *C. parviflora*, showed different features compared to other species in this genus. At the LSC/IRb and SSC/IRa border, the IR region of *C. parviflora* expanded, while at the junction of IRb/SSC, the border moved towards the IR region. These findings showed that the changes at IR/SC borders are random and very minimal, which further support the notion that chloroplast in plants evolved slowly.

### 4.2. Molecular Markers for Cardamine

Polymorphic SSRs are the same units with different unit numbers located in the homologous regions. They are frequently used to identify variable species complexes [23,34,64,65,66,67]. In this study, a total of 26 polymorphic SSRs are identified. The polymorphic SSRs identified in this study could be used as candidate genetic markers for further population genetic studies in the genus *Cardamine*. Given the variability of the regions related to these SSRs (Table 4), many of the regions were found to be mutation hotspots (Pi > 0.01), which further confirmed the concept that the presence of the polymorphic SSRs is correlated with the genome recombination and rearrangement events [66]. 

The most divergent regions among the *Cardamine* species, as determined by a comparison of nucleotide variability, were *trnH*-GUG-*psbA* (Pi = 0.06992), *ndhK*-*ndhC* (Pi = 0.04848), *trnW*-CCA-*trnP*-UGG (Pi = 0.04147), *rps11*-*rpl36* (Pi = 0.04705) and *rpl32*-*trnL*-UAG (Pi = 0.04219). The variability in these regions was much higher than that of the regions *ndhF* (Pi = 0.01355), *matK* (Pi = 0.01747), *aptB* (Pi = 0.00754) and *rbcL* (Pi = 0.00338), which were formerly used as DNA barcodes for the family Brassicaceae and other angiosperms [30,68]. The highly variable regions identified here could be validated and used as molecular markers in future species delimitation and phylogenetic studies.

### 4.3. Inferring the Phylogeny and Species Identification of Cardamine

*Cardamine* and *Dentaria* have been recognized as closely related genera by several authors. *Dentaria* is considered to be different from *Cardamine* in having larger flowers, fleshier and larger rhizomes, less-often palmately divided cauline leaves and ordinarily cotyledons [69,70,71]. However, Crantz [72] united two genera under *Cardamine* and his treatment has been followed by many authors [1,3,73,74,75,76,77,78,79]. In Schulz’s classification [73,74], the species previously published in *Dentaria* were placed into four sections, sect. *Dentaria* (L.) O.E.Schulz, sect. *Eutreptophyllum* O.E. Schulz, sect. *Sphaerotorrhiza* O.E. Schulz, and sect. *Macrophyllum* O.E. Schulz. Jones [75] divided the genus into two subgenera and treated *Dentaria* as a subgenus (subg. *Dentaria* (L.) Jones) within *Cardamine*. Based on the molecular evidence from the *trnL* intron and *ndhF* sequence data, Sweeney and Price [80] concluded that *Dentaria* is not monophyletic and the inclusion of *Dentaria* within *Cardamine* could be accepted. In the present study, seven species of *Dentaria* were included, nested within *Cardamine* and separated into four clades. This implies that the genera *Cardamine* and *Dentaria* are paraphyletic, and the broader sense of *Cardamine*, including the genus *Dentaria*, is monophyletic. Therefore, our analysis supports uniting *Dentaria* and *Cardamine* as one single genus. 

The infrageneric classification of *Cardamine* is controversial. In his monograph of *Cardamine*, Schulz [73,74] recognized 130 species and divided the genus into 13 sections. In his treatment, six sections are monotypic, and the largest section, *Cardamine*, includes nearly two-thirds of the species of the genus. Schultz’s classification has been criticized by several authors for over-emphasizing a few morphological characteristics [3,81]. Jones [75] recognized two subgenera, i.e., subg. *Cardamine* and subg. *Dentaria* (L.) Hook.f. The previous molecular studies [2,12,80,82,83] have shown that some of his sections (e.g., sect. *Cardamine*, sect. *Dentaria*, sect. *Macrocarpus* O.E. Schulz, sect. *Macrophyllum* and *Papyrophyllum* O.E. Schulz) are not monophyletic. In the present study, to follow the classification of Schulz [73,74], one species of sect. *Cardaminiella*, two species of sect. *macrophyllum*, eleven species of sect. *Cardmine*, and eight species of sect. *Dentaria* were included. Our results show that sect *Macrophyllum*, sect. *Dentaria* and sect. *Cardamine* are not monophyletic. Neither Schulz’s nor Jones’s classification is supported in the present study, which is in accord with the previous works. However, due to the limit species involved in the present studies, the infrageneric classification is not resolved and further studies are necessary to include more species. 

Understanding genetic variation within the *Cardamine* species plays an important role in improving genetic diversity and is essential for future analysis of the reproduction patterns and adaptive changes of the species within it. It could further enhance our understanding the mechanisms of selenium tolerance, which yet have only been focused on one species, *C. hupingshanensis*. The complete cp genomes have been successfully used to resolve phylogenetic relationships at multiple taxonomic levels in recent years [23,25,26,27,30,33,34,37,55,61,63,66,84,85]. Our phylogenetic results, based on 51 cp genomes of Brassicaceae, further confirmed the monophyly of the genus *Cardamine* and the placement of this genus within the tribe *Cardamineae*, which was consistent with recent Brassicaceae phylogenetic studies based on plastid or nuclear datasets [57,58,60]. In this study, all the analyzed 17 *Cardamine* species were classified into three clades. The earliest diverging lineage comprises *C. heptaphylla, C. pentaphyllos* and *C. kitaibelii*, which is characterized by flowering leafless stems. In contrast, the other two clades have leafy flowering stems. Clade 2 contained ten species: *C. oligosperma*, *C. hirsuta*, *C. macrophylla*, *C. glanduligera*, *C. tangutorum*, *C. impatiens*, *C. quinquefolia*, *C. abchasica*, *C. bipinnata* and *C. bulbifera*. This clade is characterized by cauline compound leaves. Clade 3 consisted of ten species, which have the synapomorphy of the presence of cauline leaves and erect stems, simple or branched above and/or basally. Within this clade, two samples of *C. hupingshanensis* formed a cluster with *C*. *lyrata* and *C. circaeoides*. However, the samples from Enshi (accession ON322745) is isolated with our sample from Hupingshan population from which the type specimens of *C. hupingshanensis* were made. Before *C. hupingshanensis* was published, plants from Enshi were named as *Cardamine enshiensis* by Y.Y Wu and T.Y. Xiang but not validly published. After this, *C. enshiensis* was merged with *C. hupingshanensis* by Wang et al. [86]. *C. hupingshanensis* is similar to *C. circaeoides* in terms of the simple leaves, which are rarely obscurely two- or three-lobed, and cauline leaves resemble basal leaves, but differ in their broadly obovate petals, 8–10 x 7–9 mm. Besides, *C. circaeoides* is widely distributed from Central & Western China to the Himalayan region. *Cardamine lyrata* is quite different from *C. hupingshanensis* and *C. circaeoides* due to its pinnatifid leaves. Since we were not able to examine the voucher specimens of the *C. lyrata* sample (accession MZ846206), its identification remains doubtful in this study. Our phylogenetic results suggested that *C. hupingshanensis*, *C. enshiensis* and *C. circaeoides* may form a natural species complex with some other species complexes in the presence of simple cauline leaves that resemble basal leaves, and are rarely obscurely two- or three-lobed. Our results also indicated that together with the high-selenium-tolerance plant *C. hupingshanensis*, *C. enshiensis* and *C. circaeoides* could be used as ideal plants to study the genetic mechanisms of plant selenium tolerance.

## 5. Conclusions

In this study, we sequenced the cp genome of *C. hupingshanensis* and compared the genome with other 16 *Cardamine* species. The *Cardamine* cp genomes were found to be well-conserved in genome structure, size, and gene content. The whole cp genome of *C. hupingshanensis* is presented here with 155,226 bp sequence length. The size is within the range of all previously sequenced *Cardamine* genomes. The protein coding sequences based on the complete cp genome data produced highly resolved phylogeny with strong support in this taxonomically complex group. *C. hupingshanensis* was found to be closest with *C. enshiensis* and *C. circaeoides* with strong support, suggesting that these three species may form a natural species complex. The SSRs identified in this study have advantages in single-parent inheritance. They could be used as molecular markers in future genetic diversities analysis and species identification. Furthermore, five higher-variable regions were identified, which were suitable as molecular markers for *Cardamine* species identification. In summary, the results obtained in this study should provide valuable information for future studies of the genetic diversity and the evolutionary history of *Cardamine*. In addition, our results laid the foundation for the phylogenetic analysis of *Cardamine* and the precise determination of *C. hupingshanensis*, which should contribute to further studies on the mechanisms of selenium tolerance.

## Figures and Tables

**Table 1:** Summary of eighteen *Cardamine* chloroplast genome features.

Species Name	Accession Number	Genome Size (bp)	LSC (bp)	SSC (bp)	IR (bp)	CDS Length	GC Content
*Cardamine hupingshanensis*	NC_065146	155,226	84,287	17,943	26,498	78,099	36.31%
*Cardamine macrophylla*	MF405340	155,393	84,478	17,957	26,479	79,410	36.35%
*Cardamine bulbifera*	NC_049603	155,295	84,473	17,858	26,482	79,146	36.39%
*Cardamine glanduligera*	MK637680	153,828	83,124	17,716	26,494	79,179	36.43%
*Cardamine hirsuta*	MK637681	153,934	83,228	17,782	26,462	79,002	36.41%
*Cardamine kitaibelii*	MK637684	155,160	84,238	17,886	26,518	78,759	36.36%
*Cardamine pentaphyllos*	MK637691	155,560	84,573	17,935	26,526	78,933	36.35%
*Cardamine quinquefolia*	NC_049620	155,009	84,188	17,853	26,484	79,164	36.39%
*Cardamine amariformis*	MZ043776	155,598	84,575	17,975	26,524	79,056	36.34%
*Cardamine occulta*	MZ043777	154,796	83,836	17,936	26,512	79,497	36.33%
*Cardamine fallax*	MZ043778	154,797	83,817	17,938	26,521	79,410	36.32%
*Cardamine impatiens*	NC_026445	155,611	84,696	17,949	26,483	79,395	36.33%
*Cardamine resedifolia*	NC_026446	155,036	84,151	17,867	26,509	79,518	36.30%
*Cardamine amara*	NC_036962	154,561	84,281	17,706	26,287	78,488	36.40%
*Cardamine oligosperma*	NC_036963	153,888	83,194	17,768	26,463	79,219	36.41%
*Cardamine parviflora*	NC_036964	154,684	83,934	17,732	26,509	79,621	36.36%
*Cardamine enneaphyllos*	NC_049605	155,221	84,195	18,002	26,512	73,794	36.28%

**Table 2:** List of genes encoded by the chloroplast genome of *Cardamine hupingshanensis*.

Category	Gene Groups	Name of Genes
Self-replication (60 unique genes)	Large subunit of ribosomal proteins	*rpl2*×2, *rpl14*, *rpl16*, *rpl20*, *rpl22*, *rpl23*×2, *rpl32*, *rpl33*, *rpl36*
	Small subunit of ribosomal porteins	*rps2*, *rps3*, *rps4*, *rps7*×2, *rps8*, *rps11*, *rps12*, *rps14*, *rps15*, *rps16*, *rps18*, *rps19*
	RNA polymerase	*rpoA*, *rpoB*, *rpoC1*, *rpoC2*
	Ribosomal RNA gene	*rrn4.5*×2, *rrn5*×2, *rrn16*×2, *rrn23*×2
	Transfer RNA genes	*trnA*-UGC×2, *trnC*-GCA, *trnD*-GUC, *trnE*-UUC, *trnF*-GAA, *trnfM*-CAU, *trnG-*GCC, *trnG*-UCC, *trnH-GUG*, *trnI*-CAU×2, *trnI*-GAU×2, *trnK*-UUU, *trnL-*CAA×2, *trnL*-UAA, *trnL*-UAG, *trnM*-CAU, *trnN*-GUU×2, *trnP*-UGG, *trnQ*-UUG, *trnR-*ACG×2, *trnR*-UCU, *trnS*-GCU, *trnS*-GGA, *trnS*-UGA, *trnT*-GGU, *trnT*-UGU, *trnV-*GAC×2, *trnV-*UAC, *trnW-*CCA, *trnY-*GUA
	Translational initiation factor	*infA*
Photosynthesis (57 unique genes)	Subunits of ATP synthase	*atpA*, *atpB*, *atpE*, *atpF*, *atpH*, *atpI*
	Subunits of Photosystem Ⅰ	*psaA*, *psaB*, *psaC*, *psaI*, *psaJ*, *ycf3*, *ycf4*
	Subunits of Photosystem Ⅱ	*psbA*, *psbB*, *psbC*, *psbD*, *psbE*, *psbF*, *psbH*, *psbI*, *psbJ*, *psbK*, *psbL*, *psbM*, *psbN*, *psbT*, *psbZ*
	Subunits of cytochrome b/f complex	*petA*, *petB*, *petD*, *petG*, *petL*, *petN*
	Subunits of rubisco	*rbcL*
	Subunits of NADH-dehydrogenase	*ndhA*, *ndhB*×2, *ndhC*, *ndhD*, *ndhE*, *ndhF*, *ndhG*, *ndhH*, *ndhI*, *ndhJ*, *ndhK*
Other genes (8 unique genes)	Subunit of acetyl-CoAcarboxylase	*accD*
	C-type cytochorome synthesis gene	*ccsA*
	Envelope membrane protein	*cemA*
	ATP-dependent Protease	*clpP*
	Maturase K	*matK*
	Component of TIC complex	*ycf1*
	Genes of unknown function	*ycf2*×2, *ycf15*×2

**Table 3:** Codon usage table of *Cardamine hupingshanensis*.

Amino Acid		Codon	No.	RSCU	Proportion	tRNA
Phe	1561	UUU	1054	1.35	6.00%	
		UUC	507	0.65		trnF-GAA
Leu	2768	UUA	935	2.03	10.63%	trnL-UAA
		UUG	521	1.13		trnL-CAA
		CUU	582	1.26		
		CUC	178	0.39		
		CUA	386	0.84		trnL-UAG
		CUG	166	0.36		
Ile	2260	AUU	1129	1.5	8.68%	
		AUC	411	0.55		trnI-GAU
		AUA	720	0.96		
Met	594	AUG	594	1	2.28%	trnfM-CAU, trnI-CAU, trnM-CAU
Val	1387	GUU	517	1.49	5.33%	
		GUC	178	0.51		trnV-GAC
		GUA	489	1.41		trnV-UAC
		GUG	203	0.59		
Ser	2016	UCU	566	1.68	7.74%	
		UCC	303	0.9		trnS-GGA
		UCA	420	1.25		trnS-UGA
		UCG	202	0.6		
		AGU	402	1.2		
		AGC	123	0.37		trnS-GCU
Pro	1034	CCU	419	1.62	3.97%	
		CCC	190	0.74		
		CCA	292	1.13		trnP-UGG
		CCG	133	0.51		
Thr	1328	ACU	544	1.64	5.10%	
		ACC	230	0.69		trnT-GGU
		ACA	419	1.26		trnT-UGU
		ACG	135	0.41		
Ala	1374	GCU	637	1.85	5.28%	
		GCC	216	0.63		
		GCA	372	1.08		trnA-UGC
		GCG	149	0.43		
Tyr	957	UAU	785	1.64	3.68%	
		UAC	172	0.36		trnY-GUA
His	605	CAU	458	1.51	2.32%	
		CAC	147	0.49		trnH-GUG
Gln	921	CAA	720	1.56	3.54%	trnQ-UUG
		CAG	201	0.44		
Asn	1257	AAU	962	1.53	4.83%	
		AAC	295	0.47		trnN-GUU
Lys	1479	AAA	1139	1.54	5.68%	trnK-UUU
		AAG	340	0.46		
Asp	1034	GAU	840	1.62	3.97%	
		GAC	194	0.38		trnD-GUC
Glu	1351	GAA	1028	1.52	5.19%	trnE-UUC
		GAG	323	0.48		
Cys	310	UGU	234	1.51	1.19%	
		UGC	76	0.49		trnC-GCA
Trp	448	UGG	448	1	1.72%	trnW-CCA
Arg	1536	CGU	339	1.32	5.90%	trnR-ACG
		CGC	106	0.41		
		CGA	350	1.37		
		CGG	118	0.46		
		AGA	466	1.82		trnR-UCU
		AGG	157	0.61		
Gly	1730	GGU	571	1.32	6.65%	
		GGC	163	0.38		trnG-GCC
		GGA	721	1.67		trnG-UCC
		GGG	275	0.64		

**Table 4:** The polymorphic SSRs among 17 *Cardamine* species.

SSR	*C. hupingshanensis*/*C. gladuligera*/*C. bulbifera* /*C. macrophylla*/*C. parviflora*/*C. oligosperma*/*C. amara*/*C. resedifolia*/*C. quinquefolia*/*C. impatiens*/*C. kitaibelii*/*C. pentaphyllos*/*C. hirsuta*/*C. amariformis*/*C. occulta*/*C. fallax*	Location	Regions	Pi
T	10/11/10/12/12/10/12/12/12/-/12/12/12/12/12	*matK*	LSC	0.01642
A	20/-/12/-/16/12/10/10/11/10/10/15/13/17/13	*trnK*-UUU--*rps16*	LSC	0.00639
TA	10/12/10/12/10/-/10/12/12/-/-/10/6/7/7	*rps16*--*trnQ*-UUG	LSC	0.03666
T	14/-/13/11/-/13/11/-/-/18/21/-/12/11/11	*trnR*-UCU--*atpA*	LSC	0.00000
T	11/11/11/11/11/11/12/11/11/11/11/11/11/11/11	*rpoC2*	LSC	0.00000
T	10/13/12/16/-/11/13/14/14/-/-/-/14/16/16	*rpoC1* intron	LSC	0.01720
T	12/11/15/15/12/10/10/-/15/15/15/12/15/18/19	*trnE*-UUC--*trnT*-GGU	LSC	0.03642
AT	20/20/24/18/12/14/10/18/16/10/14/12/8/10/10	*trnE*-UUC--*trnT*-GGU	LSC	0.03642
A	17/10/-/-/-/14/14/10/13/10/10/12/-/-/10	*trnT*-GGU-*psbD*	LSC	0.00000
A	12/11/11/-/16/11/14/11/11/14/14/18/-/-/-	*psbZ*-*trnG*-UCC	LSC	0.03965
T	11/16/13/17/16/15/11/15/16/16/16/15/15/15/15	*psaA*-*ycf3*	LSC	0.02436
A	11/11/12/11/-/14/14/11/12/16/21/-/-/16/15	*psaA*-*ycf3*	LSC	0.02436
T	10/-/-/14/-/-/-/10/12/11/11/-/14/14/14	*trnM*-CAU-*atpE*	LSC	0.00000
T	10/10/10/-/10/13/10/12/12/11/11/11/-/10/10	*atpB*-*rbcL*	LSC	0.01690
T	10/10/10/-/-/-/11/10/-/12/12/-/10/10/10	*rbcL*-*accD*	LSC	0.02311
T	10/10/10/10/-/10/10/10/10/12/12/-/10/10/10	*accD*	LSC	0.01055
T	10/11/10/10/10/17/10/13/-/10/10/-/11/10/10	*clpP* intron	LSC	0.01235
T	13/13/13/13/13/13/13/13/13/12/17/13/13/13/13	*rpoA*	LSC	0.02929
T	13/10/-/-/-/10/13/10/10/11/11/-/10/14/15	*rpl16* intron	LSC	0.02311
T	10/19/14/14/10/-/12/15/14/15/14/10/-/-/-	*rps12*-*trnV*-GAC	IR	0.00572
AT	16/-/-/-/12/12/10/-/-/-/-/12/8/8/8	*trnR*-ACG-*trnN*-GUU	IR	0.00000
T	12/-/15/-/11/13/-/11/-/11/11/-/11/-/-	*ndhF*-*rpl32*	SSC	0.03768
A	13/10/14/14/-/13/10/11/12/12/18/-/13/-/14	*ccsA*-*ndhD*	SSC	0.03162
T	12/12/12/13/11/12/12/12/12/12/12/11/12/10/10	*ycf1*	IR	0.00635
AT	16/12/14/-/12/12/10/12/16/-/-/12/10/8/8	*trnN*-GUU-*trnR*-ACG	IR	0.00000
A	10/19/14/15/10/-/12/15/14/15/14/10/-/-/-	*trnV*-GAC-*rps7*	IR	0.00000

LSC, large single copy; IR, inverted repeat; SSC, small single copy.

## Data Availability

The chloroplast genome of *Cardamine hupingshanensis* has been deposited into the NCBI database with the accession number NC_065146. The raw genomic data have been deposited into the NCBI SRA database with the accession number PRJNA894376.

## Supplementary Materials

The following supporting information can be downloaded at: https://www.mdpi.com/article/10.3390/genes13112116/s1, Table S1: Summary of all analyzed chloroplast genome Genbank accession numbers in Brassicaceae; Table S2: Summary of SSRs and tandem repeats numbers in all *Cardamine* species; Table S3: Simple sequence repeats (SSRs) in the cp genomes of *Cardamine hupingshanensis*.
